# Colour Selection and Olfactory Responses of *Papilio demoleus* during Foraging and Courtship

**DOI:** 10.3390/insects14030249

**Published:** 2023-03-02

**Authors:** Shunan Chen, Mingtao Li, Ji Liu, Ying Feng, Jun Yao, Lei Shi, Xiaoming Chen

**Affiliations:** 1 Resource Insect Research Center, Chinese Academy of Forestry, Kunming 650233, China; chensa@ion.ac.cn (S.C.);; 2 Key Laboratory of Breeding and Utilization of Resource Insects of State Forestry Administration, Kunming 650233, China; 3 Institute of Highland Forest Science, Chinese Academy of Forestry, Kunming 650233, China

**Keywords:** visual sensory, olfactory sensory, opsin genes, visiting flowers, courtship behaviour

## Abstract

**Simple Summary:**

In this work, we analysed the visual and olfactory sensory responses of the butterfly *Papilio demoleus* during foraging and courtship. We found that *P. demoleus* has a preference for red and orange. There is a significant difference between the visual and olfactory behaviour of both males and females; males rely more on visual cues, and females respond more to olfactory signals during foraging and courtship. Colour is the dominant factor during foraging and courtship; members of *P. demoleus* find the target by colour. Butterfly movement is an important role during the courtship of *P.demoleus*, and odour can increase accurate recognition of males and females. Opsin genes of *P. demoleus* were analysed, and we found that *P. demoleus* have genes that recognise the long wavelength, blue and ultraviolet regions of the spectrum. These pieces of evidence are beneficial to understand the coevolve of the butterfly and plant.

**Abstract:**

Colours and odours are the most important cues for butterflies to localise food and mating partners. We studied the visual and olfactory responses of the widely distributed butterfly *Papilio demoleus Linnaeus* during foraging and courtship. *P. demoleus* visited odourless flowers with six colours except green and black, with red as the favourite colour (650–780 nm). Males and females differed in behaviour while visiting flowers. Males were more active than females during foraging. The application of honey water resulted in a significant increase in flower visits by both females and males, and they scarcely visited the apetalous branches with odours. Under natural conditions, four patterns were observed: males chasing males (42.28%), males chasing females (30.56%), females chasing females (13.73%), and females chasing males (13.43%). Males chasing males was the most frequent, probably because males drive away competing con-specific males. When butterflies visited odourless mimics, males chasing females (70.73%) and males chasing males (29.27%) also occurred, indicating that males could accurately distinguish mates using colours only without any chemical cues, and females need chemical cues. The behavioural responses of *P. demoleus* to floral visits and courtship suggest that colour is the dominant factor during foraging and courtship. We verified the presence of *P. demoleus* rhodopsin genes, including Rh2, Rh3, Rh4, and Rh5, for long wavelength, blue, and ultraviolet (UV) spectrum recognition, which is consistent with the colour recognition of flowers and wings during visiting flowers and courtship.

## 1. Introduction

Flowering plants provide floral information, such as odour, colour, size and shape, that is important to butterfly’s foraging and mating [1,2,3,4], and butterflies use floral information to recognise flowers and rewards [5,6,7]. The colour and odour of flowers seem to be the most important cues during foraging. Butterflies use colour cues to find food via colour vision [5,8,9,10,11,12]. Odours also serve as important cues for butterflies [1,13]. Specific volatiles of flowers can stimulate sensory responses, orientation, and foraging behaviour in insects [5,7,14]. In natural habitats with a variety of plant species, the flexibility of foraging preferences of butterflies has clear adaptive value, enabling butterflies to effectively track the most valuable resource. Different butterfly species feed on the nectar of different plants [15]. For example, *Pieris napi* and *P. rapae* have a preference for *Vicia cracca* L. and *Lotus corniculatus* L. [16] and would constantly visit these plants if they provided sufficient nectar [3]. Increasing the stability of flower resources reduces the time required to search for nectar, which increases the time available for other important activities, such as reproduction. When preferred nectar plants are insufficient, butterflies resort to foraging on other valuable plant species that provide nectar [2,17]. However, butterflies focus on one plant during foraging [18]. The importance of colour vision in detecting flowers has been demonstrated in different butterflies. For example, *P. napi* showed a flexible choice of flower colours [3]. Different butterfly species use colour and odour cues in different ways [1,13]. According to the relative usage of olfactory and visual sensory cues during foraging, butterflies are divided into four types: those using mainly visual cues with olfactory cues as supplementary cues, those using mainly olfactory cues with visual cues as supplementary cues, those using both visual and olfactory cues equally, and those relying only on olfactory cues [19]. Butterflies can distinguish between flowers of different shapes and sizes, and large butterflies usually preferentially visit large flowers [19]. A combination of innate and acquired behaviours in butterflies is used to find flowers [4].

Sexually mature butterflies use visual and olfactory signals to distinguish other species and look for a mate during courtship [20]; both visual and olfactory cues are important for finding and distinguishing mates during courtship [21]. Butterfly species with sexual dimorphism, i.e., phenotypically distinguishable males and females, have significantly different colour characteristics [22,23]. The recognition of individuals in butterflies is primarily dependent on wing colouration, including ultraviolet (UV) reflection patterns and wing stripes [24]. For example, *Colias eurytheme* females use differences in UV reflection patterns between males of different species to distinguish intraspecific males [25]. Similarly, *Laodamia subattributes* distinguish their own species from other species using visual cues, such as wing colours [24]. On the other hand, volatile chemicals are also often used to distinguish males and females [21,25,26], and females can also accept or reject males on the basis of volatiles during courtship [27]. Butterflies also use volatile chemicals, searching for host plants to lay eggs on to provide their larvae with easily-accessible nearby food. Butterflies’ feeding habits are divided into euryphagy and oligophagy, which could be related to their distinguishing ability to chemical volatiles of hosts, inhabit, and evolve [28,29,30]. *Cethosia cyane* males depend on visual and olfactory cues to distinguish between males and females during courtship [26]. Moreover, males use volatiles to distinguish between mated and unmated females [31]. *P. napi* males transfer their own synthesised methyl salicylate (MeS) to females during mating, and when the female is courted by other males, these volatiles are released to deter other males from mating with mated females [27].

Colours play an important role in the evolution of butterflies, but how species-specific colour recognition mechanisms evolved remains a mystery. Habitat and biological characteristics such as foraging and courtship behaviour affect the structure and function of the butterfly’s visual system [12,32]. Many studies have shown that insects have three or four photoreceptors allowing them to perceive colours ranging from UV (300 nm) to red (700 nm). The visual system of butterflies is more diverse than the highly conserved trichromatic visual system of hymenopterans [33]. For example, *Papilio xuthus* has six different spectral receptors, namely, UV, violet, blue, green, red, and a broad-band-sensitive receptor [9,34]. The opsin genes Rh1, Rh2 and Rh3 can recognise the long-wavelength spectrum, Rh4 (BL) recognises the blue spectrum, and Rh5 can recognise the UV spectrum [9,34]. Moreover, *P. maackii* has at least four spectral receptors [33].

The interaction of visual and olfactory cues in regulating butterfly behaviour is poorly understood, and studies describing sensory differences among butterfly species are limited. *P. demoleus* Linnaeus is mainly distributed in Yunnan, Guizhou and Sichuan and other regions in China and has important ornamental and ecological value because it is the main pollinator of some economically important plants [35]. The mechanisms of visual and olfactory functions used by *P. demoleus* remain unclear. In this study, we observed colour recognition behaviours during foraging and courtship, compared the effects of colour, odour, and the combined signals of colour and odour, and analysed the influence of flight movement on butterfly courtship. We also analysed the visual and olfactory patterns used by *P. demoleus*, and the types of opsin genes in the compound eye of *P. demoleus* were detected. Finally, we discuss the ecological significance of visual and olfactory cues during foraging and courtship. These pieces of evidence are beneficial to understand the coevolve of the butterfly and plant.

## 2. Materials and Methods

### 2.1. Experimental Facility

The observations were carried out at the Middle Yunnan Plateau Experimental Station, The Research Institute of Resources Insects, Chinese Academy of Forestry, Kunming Yunnan, China (25°3′48″ N, 102°3′48″ E). The elevation of the location is 1808 metres above sea level, with an annual average temperature of 16.2 °C and average rainfall of 930~950 mm. The location has a subtropical plateau monsoon climate. The experiment was carried out in a transparent mesh net house with dimensions of 8 m (long) × 8 m (width) × 4 m (high).

### 2.2. Butterflies

*P. demoleus* butterflies were maintained in a greenhouse. Butterfly eggs were collected from the host plants of *P. demoleus* in the Butterfly Garden of the Chinese Academy of Forestry and incubated into larvae in a breeding cage, and larvae were fed into pupae with the leaves of the host plants. When larvae of *P. demoleus* became pupae, the pupae were placed in a growth chamber for emergence (temperature: 28 °C; humidity: 70%; light period: 12 h/24 h). Three days after the eclosion, the butterflies were used for observation.

### 2.3. Artificial Flowers and Apetalous Branch

**Figure 1:**
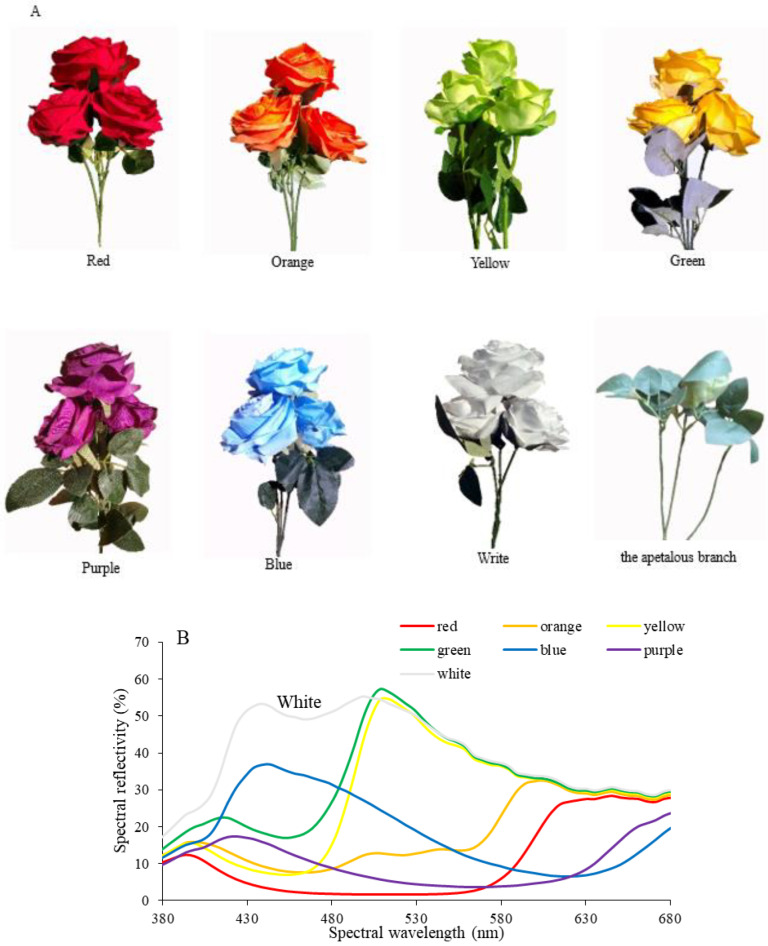
Artificial flowers and their spectral reflectance. (**A**). Artificial flowers with seven colours and an apetalous branch. (**B**). Spectral reflectance of the seven colours.

Artificial flowers. Artificial flowers were produced from cotton cloth with 8 colours, namely, red, orange, yellow, green, blue, purple, white (Figure 1A) and black. The flowers had similar corolla dimensions, with a diameter of 8.56 ± 0.18 cm and a depth of 2.73 ± 0.04 cm. The artificial flowers were washed, the spectral reflectance of the corolla of artificial flowers (Figure 1B) of the flowers was measured using a spectrograph, and the artificial flowers were positioned within the experimental arena, as described in more detail in a previous study [36]. The spectral reflectance of the black flowers was 0.

The apetalous branches. The corolla was removed from the artificial flowers of 8 colours, leaving only apetalous branches (Figure 1A).

### 2.4. Butterfly Mimics

Butterfly mimics. Artificial butterfly wing mimics were made from plastic materials with colours similar to those of real butterflies. Empty mimics. Colourless plastic mimics of butterflies were also similarly made and used as controls. After ensuring that the wing mimics did not release any volatiles, they were used for subsequent observations of butterfly courtship behaviour.

Odours of females or males. Fresh dead female and male butterflies were immersed in a solution of n-hexane100 μg/μL for more than 12 h to extract insect sex pheromones according to the conventional method [37,38,39]. The extract was filtered through filter paper and then sprayed extract on mimics for observation of the butterflies’ chase behaviour. The spectral reflectance of butterfly wings. Spectral reflectance measurements of male and female butterfly wings were performed according to previous research methods [36].

### 2.5. Observation of the Behaviour of Butterflies Visiting Flowers

Three days after eclosion, healthy butterflies were collected, and 20 males and 20 females were selected for each experiment per day, using a different butterfly group every day. Each experiment was repeated for 3 days from 8:30 a.m. to 18:30 p.m.

The female and male butterflies labelled with Arabic numerals on the wings were released to the net house to observe the frequency of visits to flowers of different colours. Artificial flowers with either red, orange, yellow, green, blue, purple or white colours were put in a net house. In each assay, 5 flowers of the same colour were bundled together to form a bunch, which was inserted into a transparent plastic bottle. Flowers were placed in a net house at a height of 100 cm. Different bunches of flowers were placed 200 cm apart. The positions of different flowers were rotated every 30 min.

Experiment 1. Odourless flowers with 8 colours were placed in a net house. Female and male butterflies (20 males and 20 females) were put in the net house to observe the frequency of visits to flowers with different colours.

Experiment 2. Butterfly responses to colour plus honey water during foraging. We used honey water represents the volatile organic chemicals (VOCs) of flowers because it has common VOCs in the nectar of flowers [40,41]. A 10 mL aliquot of 10% honey solution was uniformly sprayed onto each flower bunch every 30 min. Female and male butterflies (20 males and 20 females) were placed in the net house for observation.

Experiment 3. The corolla was removed from all artificial flowers, leaving apetalous branches. Female and male butterflies (20 males and 20 females) were placed in the net house for observation, and the responses of female and male butterflies to apetalous branches were recorded.

Experiment 4. The corolla was removed from all artificial flowers, leaving apetalous branches. A 10 mL 10% honey solution was uniformly sprayed onto each apetalous branch bunch every 30 min. Female and male butterflies (20 males and 20 females) were put in the net house for observation, and the response of female and male butterflies to the apetalous branches with added honeydew was recorded.

### 2.6. Observation of Courtship with Natural Butterflies and Mimics

Three days after eclosion, healthy butterflies were collected, and 10 males and 10 females were selected for each experiment per day, using different butterfly individuals for the experiment every day. Each experiment was repeated for 10 days.

The female and male butterflies were labelled with Arabic numerals on their wings. Five female wing mimics and 5 male wing mimics were placed into the net house, and 2 adjacent mimics were 100 cm apart. Colourless plastic butterfly mimics (without wings), *Danaus chrysippus* and *D. chrysippus* mimics were used as the control for the experiment (each mimic placed 10 cm apart).

Experiment 1. Natural population. Twenty sexually mature, unmated adults (10 males and 10 females) were released into a net house for observation. The numbers of chase and courtship behaviour were observed and recorded. Mated butterflies were removed immediately when we observed mating, and the same number of new individuals was added at the same time.

Experiment 2. Ten wing mimics of *P. demoleus* (5 female mimics and 5 male mimics), ten *D. chrysippus* mimics (5 female mimics and 5 male mimics) and 5 colourless plastic butterfly mimics were placed into a net house. Males or females of *P. demoleus* (10 males or 10 females) were released into the net house for observation, respectively. Female or male butterflies that chased motionless mimics and motional mimics were recorded.

Experiment 3. The materials and methods were the same as in Experiment 2; we used 10% butterfly extract by n-hexane solution and n-hexane solution as control, 2 solutions (10 μL/each solution) sprayed on mimics, sprayed them on each mimic every 30 min. 10 Males and 10 females of *P. demoleus* were released into the net house for courtship observation, and female and male butterflies chasing odourant mimics were recorded for comparing and analysing butterfly’s behaviour response to seek sex partners based on colour and odour information.

Motionless mimics remained immobile. For the motional mimics, the observer pulled the line suspending each mimic every 5 min during the experiment.

Each experiment of butterfly courtship or chase of mimics was performed for 10 days from 8:30 a.m. to 18:30 p.m. A response was recorded when butterflies chased butterflies, and butterflies chased or touched the mimics.

### 2.7. Opsin Cloning

DNA extraction: Genomic DNA was extracted from the retina of the compound eye of *P. demoleus* by DNAiso Reagent (TaKaRa, Beijing, China) and stored at −20 °C for later use.

Primer design: Referring to the gene sequences of *P. xuthus* opsins in the NCBI database (Table 1), Primer 5.0 software was used to analyse the gene sequences of opsins and design primers for PCR amplification.

PCR amplification: DNA of *P. demoleus* was used as the template, and specific primers were designed for the Rh1, Rh2, Rh3, Rh4 and Rh5 genes (Table 2). PCR was performed according to the instructions of the PrimeSTAR HS DNA Polymerase Kit (TaKaRa) with the following:

PrimeSTAR HS DNA Polymerase (TaKaRa): 50 μL, 5 × PrimeSTAR Buffer (Mg^2+^ Plus) 10 μL, dNTP mixture (2.5 mM each) 4 μL, PrimeSTAR HS DNA Polymerase (2.5 U/μL) 0.5 μL, template < 200 ng, Primer1 1 μL, Primer2 1 μL, and ddH_2_O UP to 50 μL.

The PCR amplification procedure was as follows: predenaturation at 94 °C for 5 min; 35 cycles of 94 °C for 30 s, 55 °C for 30 s, and 72 °C for 10 s; and extension at 72 °C for 10 min.

Agarose gel electrophoresis: PCR products were detected by 4% agarose gel electrophoresis.

Cloning sequencing: The MiniBEST Agarose Gel DNA Extraction Kit Ver. 4.0 (TaKaRa) was used to cut and collect the amplification product bands. PCR products were subcloned into the PMD19-T vector (TaKaRa) and transformed into TOP10 competent cells. Positive clones were screened by an LB plate containing ampicillin (Amp+). The M13 primer (upstream primer sequence GTTTTCCCAGTCACGAC; downstream primer sequence CAGGAAACAGCTATGAC) was used to identify the recombinant plasmid by liquid bacterial PCR, and the identified plasmid was submitted to Shenggong Bioengineering (Shanghai) Co., Ltd., for sequencing. The DNA sequences of Rh2, Rh3, Rh4 and Rh5 were obtained by splicing the correct sequences, and then blast analysis was performed with the NCBI database to determine sequence accuracy.

### 2.8. Data Analysis

The data were analysed using SPSS 18.0.

Fisher’s test was used to analyse the difference between visiting different colour flowers between artificial flowers sprayed with a honey solution and odourless artificial flowers, and the difference between the numbers of male and female butterflies chasing motionless mimics and motional mimics.

A 1-way ANOVA was used to test the differences in chasing models between male and female butterflies in the natural population and in mimics during the courtship.

A *t*-test was used to test the difference between the numbers of butterflies chasing male mimics and female mimics, and the difference between the numbers of butterflies chasing odourant mimics and odourless mimics, the difference in the numbers of flower visits between female and male butterflies.

The spectral reflectance of female and male butterfly wings was measured. A chi-square test (L-R *X*^2^_degrees of freedom_) was used to detect significant differences in the wing reflectance spectra of males and females.

## 3. Results

### 3.1. Olfactory and Visual Responses of P. demoleus during Foraging

**Figure 2:**
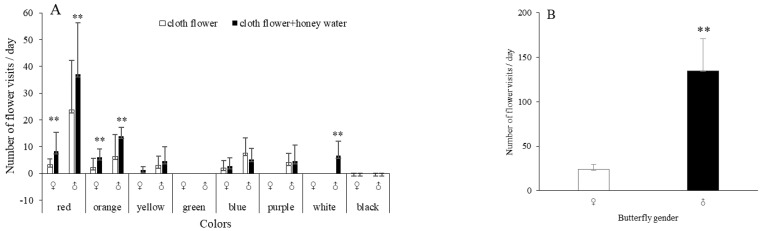

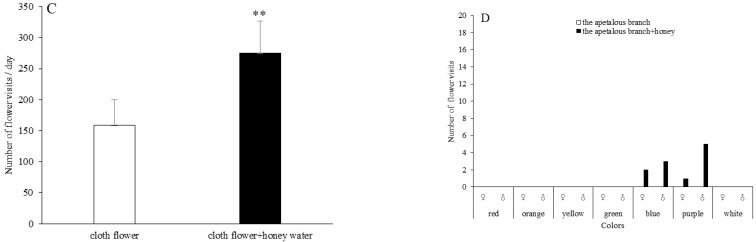
Visual and olfactory response of *P. demoleus* during visiting flowers, the number of *P. demoleus* visits was recorded. (**A**). Behaviour response of *P. demoleus* to the seven coloured artificial flowers and artificial flowers sprayed with honey water. Fisher’s test was used to analyse the pattern of butterflies visiting artificial flowers sprayed with 10% honey solution and butterflies visiting odourless artificial flowers per day. (**B**). Comparison of visiting flowers between male and female butterflies. A t−test was used to analyse the difference in the number of flower visits between female and male butterflies. (**C**). The visiting number of *P. demoleus* population to artificial flowers and artificial flowers sprayed honey water. The difference in the numbers of butterflies visiting odouless flowers and flowers supplemented with honey water in the same colour. (**D**). Behaviour response of *P. demoleus* to the apetalous branches and branches sprayed honey water. Above each bar, we provide the number of flower visits ±1 standard error. “**” indicate statistically significant differences (** < 0.01).

In the observations with artificial flowers, males and females visited all artificial flowers except for the green and black flowers. The percentages of visits to different flowers by males were 52.59% to red, 17.04% to blue, 14.07% to orange, 6.67% to yellow, 8.89% to purple and 0.74% to white flowers. For females, the visit percentages were 41.67% to red, 29.17% to orange, 25% to blue, and 4.16% to purple flowers (Figure 2A); *P.demoleus* did not visit green and black flowers. The number of visits by male butterflies was significantly higher than that of female butterflies (F = 10.434, *t* = −2.559, df = 54, *p* < 0.05; Figure 2B).

The pattern of butterflies visiting artificial flowers sprayed with 10% honey solution was consistent with that of butterflies visiting odourless artificial flowers, and the number of visiting flowers significantly increased (Fisher, *p* < 0.01; Figure 2C). The number of butterflies visiting flowers with 10% honey solution was increased by 0.73 times, female visits were increased by 1.42 times, and male visits were increased by 0.61 times. The number of male butterflies visiting red flowers was increased by 0.56 times (*n* = 6, r = 0.98, *t* = 3.374, *p* < 0.05), and that visiting orange flowers was increased by 1.21 times. Male butterflies begin to visit the white flowers. The number of female butterflies visiting red flowers increased by 1.5 times. These results indicate that odours can increase the number of *P. demoleus* visiting flowers.

Butterflies rarely visited the apetalous branches, regardless of whether they had been sprayed with the 10% honey solution (Figure 2D), indicating that *P. demoleus* mainly used colours for visual sensory recognition during foraging.

### 3.2. Visual and Olfactory Behaviour of P. demoleus during Courtship

**Figure 3:**
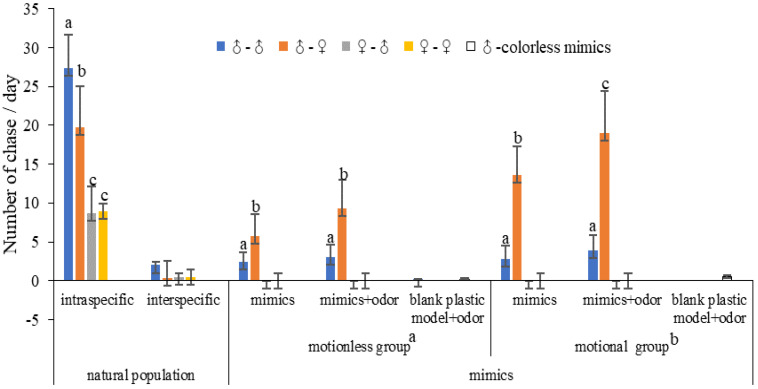
Courtship behaviour of *P. demoleus* in a natural population and mimics. Above each bar, we provide the number of chase ±1 standard error. ANOVA test was applied within each group (natural population or mimics). Fisher’s test was used to analyse the difference between the number of butterflies chasing motionless mimics and motional mimics. Different letters indicate statistically significant differences between groups (*p* < 0.05).

**Natural population.** Among our observations, events of males chasing males accounted for 42.28%, males chasing females accounted for 30.56%, females chasing females accounted for 13.73%, and females chasing males accounted for 13.43% (F = 44.078, df = 3, *p* < 0.01). Males showed significant differences in their courtship of different sexes, but females showed no significant differences, chasing males and females equally (Figure 3). During the courtship of natural butterflies of *P. demoleus*, males always took the initiative, males and females rarely chased interspecific butterflies, and individuals could distinguish between interspecific and intraspecific butterflies.

**Behavioural response of butterflies to mimics.** During the courtship of butterflies displayed towards the mimics, only males chased females (70.73%), males chased males (29.27%), and females did not chase mimics. Males were able to distinguish females and males by wing colours (F = 3.64, *t* = −3.327, df = 18, *p* < 0.01; Figure 3), and males and females did not chase interspecific mimics or blank mimics, indicating that wing colours were the main cues used during *P. demoleus* courtship.

**Behavioural response of butterflies to mimics plus odour.** After the extract of females or males and the control solution were sprayed onto the mimics, butterflies hardly chased mimics that were sprayed with the control solution, only chased mimics that sprayed extract; there are two ways: males chased males, and males chased females. Males that chased motionless and mobile mimics were significantly increased compared with those that chased the odourless mimics (*n* = 40, r = 0.857, *t* = −4.125, *p* < 0.01). Males chasing females increased by 60.34%, and males chasing males increased by 29.17% (F = 13.318, df = 3, *p* < 0.01) during chasing of the motionless mimics, males chasing females increased by 39.71%, and males chasing males increased by 39.29% (F = 43.698, df = 3, *p* < 0.01), and the behaviour of males chasing empty mimics appeared (Figure 3). The combination of colour and odour provides stronger cues than either alone for butterfly courtship.

**Behavioural response of butterflies to motional mimics.** The number of male butterflies chasing the motional mimics was significantly greater than that chasing the motionless mimics (Fisher, *p* < 0.05), and the male butterfly response to the odourant-treated mimics was stronger than that to the odourless mimics (Figure 3). The pattern of mimic recognition by female and male butterflies was similar to that observed for natural population courtship; individuals could accurately recognise female and male individuals, and the flight movement of butterflies could enhance courtship.

### 3.3. Colour and Reflectance Spectra of Wings

**Figure 4:**
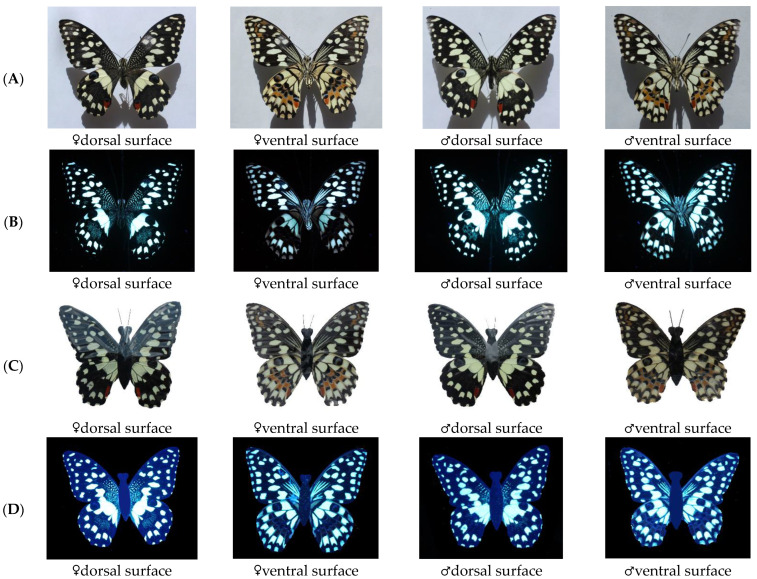
Images of females and males of natural butterflies and mimics under sunlight or ultraviolet light. (**A**). Images of butterflies under sunlight. (**B**). Images of butterflies under ultraviolet light. (**C**). Images of butterfly wing mimics under sunlight. (**D**). Images of butterfly wing mimics under ultraviolet light.

Under natural sunlight, the wings of *P. demoleus* showed a range of colours. The overall common background colour accounted for more than 50% of the wing area, and light yellow was a supplementary colour covering approximately 30% of the wing area. The dorsal front wing was mainly black (≥85%), and many yellow dots covered the surface of the base area. A stripe of circular spots covered the edge of the wing surface. There were many irregular patches of different sizes in the central region of the front wings. The dorsal surface of the hind wings was mainly black (≥60%), with yellow-brown patches, blue-purple patches, and red and black circles with blue-purple semicircles all over the leading edge of the hind wing. The ventral surface of the hind wings was mainly light yellow (≥60%), and there were several light-yellow spots at the base. Large red patches were distributed at the end of the inner edge, and the overall patch patterns were similar to those on the dorsal side (Figure 4A).

The dorsal and ventral sides of the wings of both females and males also showed rich patterns under UV light, indicating that the wings have a strong ability to reflect UV light (Figure 4B). On the integument of the butterflies, black was mixed with light-yellow colours on the dorsal side, and light-yellow colour was distributed all over the ventral surface. The ventral surface of the integument of butterflies showed strong UV reflectance. Under natural sunlight and UV light, the pattern of wing colour variation of female and male butterfly mimics was similar to that of female and male butterflies.

**Figure 5:**
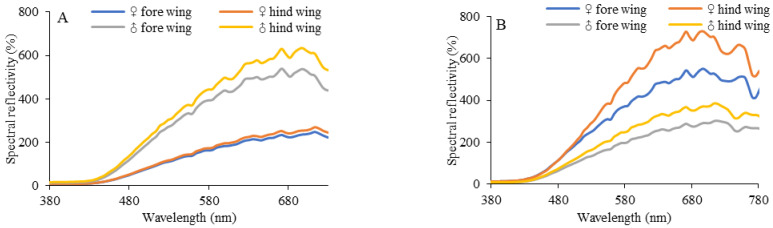
The reflectance of butterfly wings. (**A**). Dorsal surface of butterfly wings. (**B**). Ventral surface of butterfly wings. A chi-square test (L-R *X*^2^_degrees of freedom_) was used to detect significant differences in the wing reflectance spectra of males and females.

Spectrum analyses showed that the spectral reflectance of the wings of females and males at 380~780 nm increased as the wavelength gradually increased (Figure 5). Both female and male wings showed marked differences between the dorsal side and ventral side (Pearson L-R *X*^2^ = 20,575.368 ^a^, df = 1, *p* < 0.01). The spectral reflectance of the dorsal wing of males (Pearson L-R *X*^2^ = 24.253A, df = 1, *p* < 0.01) was significantly higher than that of females, and the spectral reflectance of the ventral wing of females (Pearson L-R *X*^2^ = 2.133A, df = 1, *p* > 0.05) was significantly higher than that of males. The images of females and males were significantly different.

### 3.4. Opsin Gene Analysis of P. demoleus

**Figure 6:**
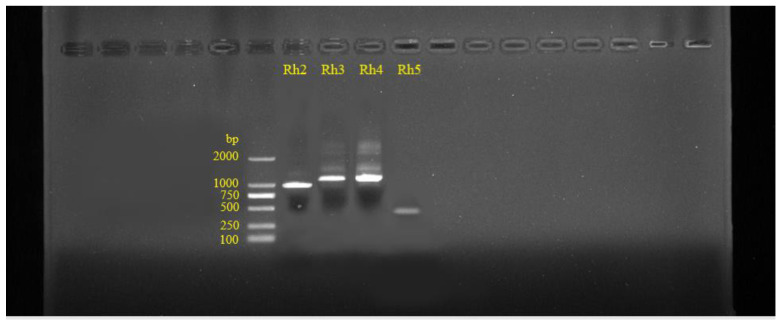
Electrophoresis map of the PCR products of opsin Rh2, Rh3, Rh4 and Rh5. Left yellow number is DNA Marker (100–2000 bp).

Five opsin genes of *P. xuthus* have been found. We detected the opsin genes in our butterflies. Four opsin genes (excluding Rh1) of *P. demoleus* were detected (Figure 6). Genes Rh2, Rh3, Rh4 and Rh5 were highly similar to opsin gene sequences in the NCBI database (Table 3). *P. demoleus* has four opsin genes, Rh2, Rh3, Rh4 and Rh5, indicating that *P. demoleus* has a wide range of spectrum recognition, can recognise a variety of colours, and can also recognise intraspecific and interspecific individuals by UV reflectance.

## 4. Discussion

*P. demoleus* butterflies have significant colour selectivity during foraging. Males preferred visiting red (52.59%), blue (17.04%) and orange flowers (14.07%), and females visited red (41.67%), orange (29.17%) and blue (25%) flowers. These behaviours are highly consistent with the four opsin genes in *P. demoleus*. When honey water was added to the artificial flowers, the flower visits of *P. demoleus* increased, indicating that odours can promote butterfly visits to flowers during foraging. In addition, the gustatory stimulus of butterflies could intensify their impression of food upon contact with the honey, causing the number of flower visits to increase. *P. demoleus* rarely visited apetalous branches, indicating that colour was the main factor affecting foraging. Male butterflies were more active than female butterflies during foraging, which may be because males need more energy during foraging.

During courtship of the natural population of *P. demoleus*, butterflies exhibited behaviours of males chasing females, males chasing males, females chasing males and females chasing females. The frequency of males chasing males was the highest, the total number of males chasing females was the second highest (≥30), and males took the initiative. During the courtship of butterflies from the natural population by *P. demoleus*, males always took the initiative. Males chasing males is driving other males and competing for mating rights.

During the courtship of mimics, the frequency of males chasing females (70.73%) was greater than that of the other categories, and males could recognise both males and females according to their wing colours. In natural populations, males chasing males was the most frequent type of behaviour. The combination of colours and male odours may be what causes males to chase. Males chasing males represents intraspecific mating competition, and males chasing males may compete for mating resources. Males will actively reject other males and compete for mating rights during courtship. Males actively reject individuals of the same sex and compete for mating opportunities. The decreased frequency of males chasing males may be due to the lack of odour cues during butterfly chasing of mimics. During butterfly chasing mimics with odours, the frequency of males chasing males dropped dramatically, while the number of males chasing females was higher. Suggested that odour plays an important role in individual recognition during butterfly courtship.

During the courtship of odourless mimics by *P. demoleus* butterflies, the behaviour of the butterflies was similar to that of *C. cyane* [26], and males were able to accurately distinguish between females and males. The frequencies of butterflies chasing butterflies and butterflies chasing odourant mimics were significantly higher than that of butterflies chasing odourless mimics, indicating that butterflies can preliminarily recognise females and males using wing colours and then accurately distinguish them by volatile organic compounds. The role of odours is also very important during butterfly courtship, and males can more accurately recognise females through odours than through colours, thus promoting successful courtship. *P. demoleus* mainly used visual sensory cues during foraging, and olfactory sensory cues promoted accurate recognition. In this experiment, the frequency of males chasing motional mimics was significantly higher than that of males chasing motionless mimics. During courtship in natural butterfly populations, butterflies usually chase males or females in flight, and the number of *P. demoleus* chasing motional mimics was greater than that chasing motionless mimics.

The courtship behaviour of *P. demoleus* and previous studies have shown that males actively recognise females and initiate courtship through multiple cues [22,24,31,42,43,44,45], and wing colours [24,25,37,46] are the main visual cues. The process by which butterflies recognise individuals of the opposite sex is quite complex. Both the colour and odour of butterflies impact courtship and different butterfly species have different visual and olfactory preferences [2,21,28,47]. The spectral reflectances of male and female wings were significantly different, and the colour of the wings also showed brighter patterns under UV light. It is suggested that the spectral reflection and UV colour of butterflies play important roles during courtship.

The Rh2, Rh3, Rh4 and Rh5 genes are expressed in the compound eyes of *P. demoleus* contain. The Rh2 and Rh3 genes may mainly target receptors of long wavelengths, such as red, orange and yellow. Rh4 recognises the blue spectrum, and Rh5 is associated with receptors of the UV spectrum [9,12,42], which is consistent with the recognition of colours and the UV spectrum of *P. demoleus*. *P. demoleus* recognised red, orange, yellow, blue, purple and white flowers during foraging, but *P. demoleus* did not visit green and black flowers or the green apetalous branches, indicating that *P. demoleus* does not use green and black as a cue for food. Red, orange, yellow, blue, purple, white, and black are the colours on the wings of each male and female of *P. demoleus*. Thus, *P. demoleus* can recognise colours similar to the colours of the wings. The results suggested that the diversity of visual gene expression underlies the recognition of diverse colours in *P. demoleus*, and the colour recognition ability of *P. demoleus* may be related to the richness of wing colours.

During evolution, butterflies can find food by the colour of flowers and identify male and female individuals by wing colours. Odours can promote butterfly foraging and mating. Butterflies possess visual genes that function in accordance with their colour-recognition behaviour. Colour may promote visual development and wing colour evolution. Butterflies finding food through flower colour promotes their visual development, and their wings become colourful. Colour vision and wing colour are coevolving and may be the main reasons for the colourful wings of butterflies.

## Figures and Tables

**Table 1:** Opsin gene information.

	Reference Opsin Gene Name	GenBank ID	Recognition Wavelength Range of the Spectrum
1	*Papilio xuthus* PxRh1 mRNA	AB007423.1	Long wavelength
2	*Papilio xuthus* PxRh2 mRNA	AB007424.1	Long wavelength
3	*Papilio xuthus* PxRh3 mRNA	AB007425.1	Long wavelength
4	*Papilio xuthus* PxRh4 mRNA	AB028217.1	Blue
5	*Papilio xuthus* PxRh5 mRNA	AB028218.1	Ultraviolet

**Table 2:** Primer information.

Opsin Gene of *P. demoleus*	Primer Name	Sequence
Rh1	Primer 1	CTTCCTGCCGAGGTAGAA
	Primer 2	CTCCGTTGATGCTCATTGG
Rh2	Primer 1	CGGCGTCTTAGGCTTCATATC
	Primer 2	AGCAATAGTCCAGGCGAGAG
Rh3	Primer 1	TGCGGTGGTTCCTTATATGGTA
	Primer 2	CGATGATGTAACTGCGGCTAA
Rh4	Primer 1	CGTTGTGCCACTACTCAC
	Primer 2	CGAGCAAGTTGTCAGGAA
Rh5	Primer 1	GAGCAGTCAGTCAGTTGGTG
	Primer 2	TACAAGCGTTGGTCAGTCC

**Table 3:** The blast results of *P.demoleus* opsins.

Opsin Gene of *P. demoleus*	Similar Genes Name	GenBank ID	Per.Ident
Rh2	PREDICTED: *Papilio polytes* opsin-1 (LOC106107962), mRNA	XM_013288994.1	92.78%
	*Papilio anactus* PaL2 mRNA for long wavelength-sensitive opsin 2, complete cds	AB725229.1	90.72%
Rh3	PREDICTED: *Papilio polytes* opsin-1-like (LOC106105734), mRNA	XM_013286167.1	94.24%
	*Papilio xuthus* PxRh3 mRNA for opsin, complete cds	AB007425.1	93.01%
Rh4	*Papilio xuthus* PxRh4 mRNA for blue opsin, partial cds	AB028217.1	88.37%
	*Papilio anactus* PaB mRNA for B-sensitive opsin, complete cds	AB725227.1	87.37%
Rh5	PREDICTED: *Papilio xuthus* opsin, ultraviolet-sensitive-like (LOC106116057)	XM_013309794.1	91.59%
	PREDICTED: *Papilio polytes* opsin, ultraviolet-sensitive-like (LOC106103556)	XM_013283340.1	91.59%

## Data Availability

Data are available on request from the authors.
